# An International System of Electro-Therapeutics

**Published:** 1895-06

**Authors:** 


### BOOK NOTICES.

AN INTERNATIONAL SYSTEM OF ELECTRO-THERAPEUTICS: for Students, General Practitioners, and Specialists. By Horatio R. Bigelow, M. D.; and thirty-eight Associate Editors. Thoroughly illustrated. In one large royal octavo volume, 1160 pages, extra cloth, $6.00 net; sheep, $7.00 net; half-Russia, $7.50 net. Philadelphia: The F. A. Davis Co., publishers, 1914 and 1916 Cherry Street.

This valuable manual has lain upon our table too long already, waiting for review. So excellent a book should have been called to the attention of the profession sooner than we have found it convenient.

It contains the very latest information upon electricity and electrical appliances, and the latest methods of electrically treating diseases of the alimentary tract, of the liver and kidneys, gout, and rheumatism.

The method of electrically treating diseases of the lungs and heart, the uterus and uterine appendages, the displacements of the uterus, the disorders of menstruation, the management of cancer and various tumors, facial blemishes, skin diseases, diseases of the nose, larynx, pharynx, and eyes, all are here minutely described each by a specialist who devotes his time exclusively to that one line of treatment. The book is profusely illustrated so that perfectly clear ideas are obtained of all apparatus described. The preface is in itself exceedingly interesting, and contains quite an exposition of the wonderful discoveries of Nikola Tesla's "high frequency currents." These phenomena are quite difficult of explanation, especially to such as have not made a special study of electricity. In this preface a very good general idea of them can be acquired by careful reading. For such as may wish an elementary idea of the Tesla discoveries, and yet have not the time to study the subject in detail the editor of this journal offers the following explanation: If a common horse-shoe magnet be held in the hand, and a common nail suspended by a thread be dangled in front of its poles, the nail will be attracted to the magnet at whatever point it be held. This shows that lines of force are reaching out from the magnet in every direction. If, now, the nail be held firmly in the hand, and passed through the air before the poles of the magnet forward and backward repeatedly, so as to cut these lines of force, electrical currents will be generated. As the nail approaches the poles the current will be momentarily in one direction and will die out entirely, as the nail arrives in front of the poles. It will be revived again in the opposite direction, the moment the nail passes the poles into the space beyond. As the nail comes back toward the experimenter, these phenomena will again be reversed. If a coil of wire, silk covered or otherwise insulated, be brought near, these currents will locate themselves in the wire and can be made to spark. If the wire be coiled about the nail, the phenomena are more apparent.

If now we imagine the nail bent round into a circle, provided with spokes and an axle, and so converted into a wheel, and the wire wrapped around the rim of the wheel in a series of disconnected spools; if we further conceive the two ends of each coil of wire soldered together and made fast to the axle, to a small plate placed longitudinally to it, we shall then have a wheel whose rim is studded with small spools of wire, whose spokes are burdened with loops running down to the axle, and whose axle is covered with a series of small plates parallel to each other completely encircling it like the staves of a barrel. From these plates the current is drawn off by slips of brass pressing upon them. This wheel, so equipped, is now placed between the poles of the magnet and allowed to revolve freely either by hand-power, or, if large enough, by a steam-engine. Such a machine is called a dynamo and is the type upon which all dynamos are constructed. Such a dynamo will give a current flowing only in one direction. If, however, no loops are made the current alternates, as previously explained, first in one direction and then in the other. Such *alternating* currents are very powerful and dangerous. They leave the wire, penetrating even the thickest insulating coverings, and do considerable damage. They are the kind of currents generated by one celebrated electric light company and because of the damage they do, the Legislature of the State of New York was petitioned to pass a law forbidding their use. At this juncture Nikola Tesla appeared upon the scene with his momentous discoveries. The alternations of current in direction were at the rate of probably one hundred to four hundred changes in a second of time. Tesla, by appropriate means, made these changes or alternations five hundred thousand, one million, and even two millions per second. As previously stated, the slower rate of alternation was dangerous and even deadly. When the alternations were at the extraordinory rate given by Tesla, they were found to be perfectly harmless to the human body. The writer of this review has seen these powerful currents passed into the body of an experimenter, who then grasped an incandescent lamp that was lying on a neighboring table, when the lamp immediately glowed strongly, so that it was possible to read by a lamp that was supplied exclusively by electricity passing through a human being! The explanation of the freedom from danger under such circumstances can be found by the analogy of a carriage wheel and its effect upon the eye. When the wheel begins to revolve all the spokes are perfectly distinguishable to the eye. As the speed of the wheel increases, the spokes become more obscure, until at its highest speed no spokes can be seen at all. Thus the eye is incapable of perceiving motion at this high speed. So it is with the effect of electricity on the human body. Low-frequency currents are perfectly perceptible to the nervous system, just as the spokes in the slowly-rotating wheel are perceptible to the eye. These currents can produce pain, disintegration, and death. When the frequency rises to the phenomenal height of one million, then the nervous organization of man can take no note of them just as the eye fails to perceive the individual spokes in the swiftly-revolving wheel, and so no damage results.

Much more might be said on this theme, but this review is already too long and not sufficiently descriptive of the volume. All we can say is that we cordially recommend the book as the latest and best exposition of the subject.

